# Perspectives of health workers engaging in task shifting to deliver health care in low-and-middle-income countries: a qualitative evidence synthesis

**DOI:** 10.1080/16549716.2023.2228112

**Published:** 2023-07-11

**Authors:** Karen Coales, Hannah Jennings, Saima Afaq, Aatik Arsh, Mujeeb Bhatti, Faraz Siddiqui, Najma Siddiqi

**Affiliations:** a Department of Health Sciences, University of York, York, UK; b Department of Health Sciences, Hull York Medical School, University of York, York, UK; c Institute of Public Health & Social Sciences, Khyber Medical University, Peshawar, Pakistan; d Department of Health Sciences, University of York, York, Uk

**Keywords:** Healthcare, LMICs, low-resource settings, review, nurse, maternal, child, HIV, mental health, non-communicable diseases, communicable diseases

## Abstract

**Background:**

Low- and middle-income countries (LMICs) are experiencing growing demand for healthcare services yet face a persistent shortage in access to specialist health workers (SHWs). Task shifting is an approach used to address this gap in service provision. Specific healthcare tasks are shifted to other, larger cadres of non-specialist health workers (NSHWs), including lay health workers with SHWs potentially taking on supervisory roles. Previous studies demonstrate that task shifting is both clinically and economically effective, however the impact of task shifting on health workers (HWs) is not fully understood.

**Objective:**

The aim of this synthesis is to generate new knowledge about what influences HWs perspectives of benefits and costs of engaging in task shifting.

**Methods:**

A qualitative evidence synthesis (QES) of peer-reviewed literature using databases CINAHL, the Cochrane Database of Systematic Reviews, Psych INFO, MEDLINE, EMBASE, Epistimonikos, Web of Science (science and social science citation index), Scopus LILACS, the African Index Medicus and Google Scholar. Eligible studies were those that included qualitative data about HWs perspectives of task shifting in LMICs. Information from eligible studies was extracted into a Google Sheet, and the data gathered were analysed thematically.

**Results:**

Fifty-four studies were included in the QES. Results were organised under three themes, ‘the cultural environment in which task shifting is employed’, ‘access to resources for task shifting’ and ‘alignment with personal values and beliefs, self-efficacy and personal emotional resilience’.

**Conclusion:**

This is the first review bringing together views about task shifting from the perspective of different cadres of HWs drawn from diverse healthcare, geographical and country settings in LMICs. Task shifting is a complex process which relies upon the active engagement of HWs. Taking into consideration factors that influence HWs perspectives, such as their personal characteristics, preparatory training, and ongoing access to resources, is important for informing how task shifted healthcare initiatives are designed and delivered to successfully widen access to healthcare in LMICs.

## Introduction

Addressing health inequalities and achieving universal health coverage for all are fundamental objectives in delivering inclusive healthcare globally [1,2]. Low- and-middle-income countries (LMICs) face great challenges in attaining equitable and accessible healthcare, not least because they face a persistent shortage in access to specialist health workers (SHWs) [3–5].

Tackling shortfalls in healthcare provision in LMICs calls for innovation in making the best use of human resources. Task shifting is one approach being utilised to address unmet health needs. Task shifting refers to the redistribution of healthcare tasks; either through the delegation of specific tasks from small cadres of SHWs horizontally to other highly qualified health workers from a different background, or more typically, vertically to larger cadres of non-specialist health workers (NSHWs), including lay health workers [1,6,7]. Historically, authors have used different terms to interchangeably to describe this process including ‘delegation’, ‘substitution’ and ‘task sharing’. However, recent discourse moves to adopt task sharing as the preferred term, distinguishing ‘task shifting’ from ‘task sharing’. Task sharing is an inclusive term that acknowledges that redistribution of tasks is often a collaborative team-based process in which teams of SHWs and NSHWs share clinical responsibility for patient care and employ iterative communication, and training to preserve high-quality outcomes [8,9]. Whilst acknowledging this important debate, in keeping with World Health Organization (WHO) definitive recommendations and guidelines for task shifting [1] and in which we have synthesised findings past studies in which a delegatory rather than collaborative process is documented, we have used ‘task shifting’ throughout for consistency.

Systematic reviews of task shifting in LMICs have found that well-designed, resourced and delivered task shifting initiatives can lead to improvement in patient health and deliver cost savings [5,7,10,11]. Task shifting works by freeing up capacity for scarce SHWs in secondary and tertiary services to be able to focus on assessing and treating patients with complex health needs as newly trained cadres of health workers (HWs) in primary and secondary health services take over responsibility for routine or specific health care tasks [1]. SHWs may take on the role of providing training, supervision and/or mentoring to NSHWs to provide support and to ensure continuity of care for patients [1].

As task shifting targets more basic, routine healthcare tasks greater numbers of NSHWs can be trained quickly and at lower cost in response to need than more expensive SHWs who typically require several years of prior profession-specific, pre-registration higher education, possibly supplemented by additional post-registration training to prepare them for their roles. NSHWs are also less expensive to employ than SHWs, opening the possibility of being able to further expand capacity to bridge treatment gaps. Consequently, this approach to addressing shortfalls in human resources is attractive to policymakers seeking to quickly respond to addressing unmet health needs and is increasingly used in LMICs across healthcare settings and programmes.

Evidence supports that with preparatory training and under SHW supervision NSHWs can effectively undertake a wide range of task shifted healthcare tasks, from health screening and prescribing medications to undertaking simple, surgical procedures and delivering specific treatment interventions such as brief psychological therapies [10,12–14]. Furthermore, task shifting offers advantages for HWs such as opportunities for professional skills development and job satisfaction [15].

However, whilst task shifting may appear as a simple solution to bridging gaps in treatment provision in low-resource settings in LMICs, successful integration into existing healthcare services is complex and challenging. The WHO global recommendations and guidelines for task shifting for HIV/AIDS [1] made 22 recommendations for task shifting encompassing adopting task shifting as a public health initiative, creating a regulatory environment, ensuring quality of care and sustainability of task shifting and organisation of clinical services. The recommendations are far reaching, requiring engagement with governmental bodies, higher education institutions and local communities as well as healthcare systems, specific healthcare services and individual healthcare workers. Poorly resourced and organised task shifting initiatives, particularly where a culture of opportunistic task shifting exists for overcoming day-to-day challenges in meeting health needs exists, are associated with detrimental outcomes for HWs and ultimately, risk compromising patient access to safe, high-quality healthcare [16]. A particular criticism of task shifting is that NSHWs typically already carry heavy workloads and have little or no capacity for shouldering extra responsibilities. In a qualitative review of task shifting programmes in Sub-Saharan countries, Mijovic et al. [17] have drawn attention to examples of task shifting being associated with poor-quality, unsafe practice, with burnout and attrition, and inequity in renumeration and access to resources comparative to their specialist colleagues, impacting NSHWs and a loss of sense of agency and diminishment of roles being reported by SHWs.

Capability of HWs is an important prerequisite for implementation and successful delivery of task shifting. Barriers and facilitators to engaging HWs in task shifting have been explored broadly through individual studies from the perspective of a range of stakeholders including policy makers, healthcare managers, patients, and community representatives and different HW cadres [18,19]. However, there is a gap in in-depth knowledge about HWs perspectives of what motivates them to engage in task shifting and what challenges and facilitators they encounter when doing so. Whilst previous qualitative reviews have sought to address this gap by synthesising findings from studies focusing on specific cadres of HW [15,17], to the best of our knowledge, this is the first synthesis aiming to bring together HW perspectives irrespective of healthcare or geographical setting and including any LMIC with the aim of determining common perspectives across cadres. Such knowledge has value in informing the design and delivery of future sustainable task shifting programmes in LMICs, particularly previously under-researched settings, taking into consideration HWs likely support needs.

## Methods

### Protocol, registration, reporting guidelines

This qualitative evidence synthesis (QES) is being reported in accordance with the reporting guidance provided in the Enhancing Transparency in Reporting the Synthesis of Qualitative Research (ENTREQ) statement [20]. The QES protocol was previously documented according to the Preferred Reporting Items for Systematic Reviews and Meta-analysis Protocols (PRISMA-P) statement [21] which ensures transparency in the formulation of findings and registered with the International Prospective Register of Systematic Reviews (PROSPERO) database at the University of York (www.crd.york.ac.uk/prospero/) on 9 April 2019 (updated on 18 November 2019 and 26 January 2023) (registration number CRD42021247132).

### Eligibility criteria

The SPIDER tool (sample, phenomenon of interest, design, evaluation, research type) [22,23] was used to facilitate the development of the research question and eligibility criteria for studies to be included in the review (see Table 1). For the purposes of the QES, task shifting was defined as delegation of any healthcare-specific procedure/intervention directly involving patients within a healthcare service or community healthcare programme. Studies that only included ancillary tasks such as administration or epidemiological data collection were excluded. The sample was limited to HWs located in LMICs as defined by the World Bank Classification of low-and-middle-income economies [24]. HWs were defined as individuals with any level of health education or training. Studies were included if HWs were employed in an existing health service, or in the case of lay HWs, where the article documented that the task shifted role constituted more than 20 hours/week and/or for which they were paid expenses. Peer-reviewed studies that included qualitative data about HWs perspectives of task shifting were eligible for inclusion in the synthesis. Studies were excluded if they were not wholly based in LMICs.

**Table 1.** Developing the research question.

SPIDER	JUSTIFICATION
S - Sample	Specialist health workers, non-specialist health workers and lay workers working in LMIC’s
Pl – Phenomenon of interest	Task shifting of healthcare specific procedures/interventions
D - Design	Interviews, focus group discussions, surveys, reflective diaries/accounts
E - Evaluation	Experiences, views, perceptions, or opinions of or beliefs about task shifting
*R* – Research type	Qualitative studies and mixed-methods studies or survey studies with qualitative elements reporting experiences and views of task shifting

### Search strategy

To identify relevant studies, databases CINAHL, the Cochrane Database of Systematic Reviews, Psych INFO, MEDLINE, EMBASE, Epistimonikos, Web of Science (science and social science citation index), Scopus, LILACS, and the African Index Medicus were searched from inception in April 2021. Reference lists of included full text studies were hand-searched and a title-only search of the first 100 results in Google Scholar was conducted to identify any other relevant studies. Searches were refreshed in March 2022. Search terms were developed for the three categories of interest ‘task shifting’, ‘health workers’ and ‘LMICs’. The search strategy was developed and piloted with the support of specialist librarians from The University of York and The University of Leeds with the list of LMICs from the World Bank [24]. A search syntax was developed (Appendix A) and adapted for each electronic database. The search was limited to studies with an English language title and abstract.

### Study selection

**Figure 1.**
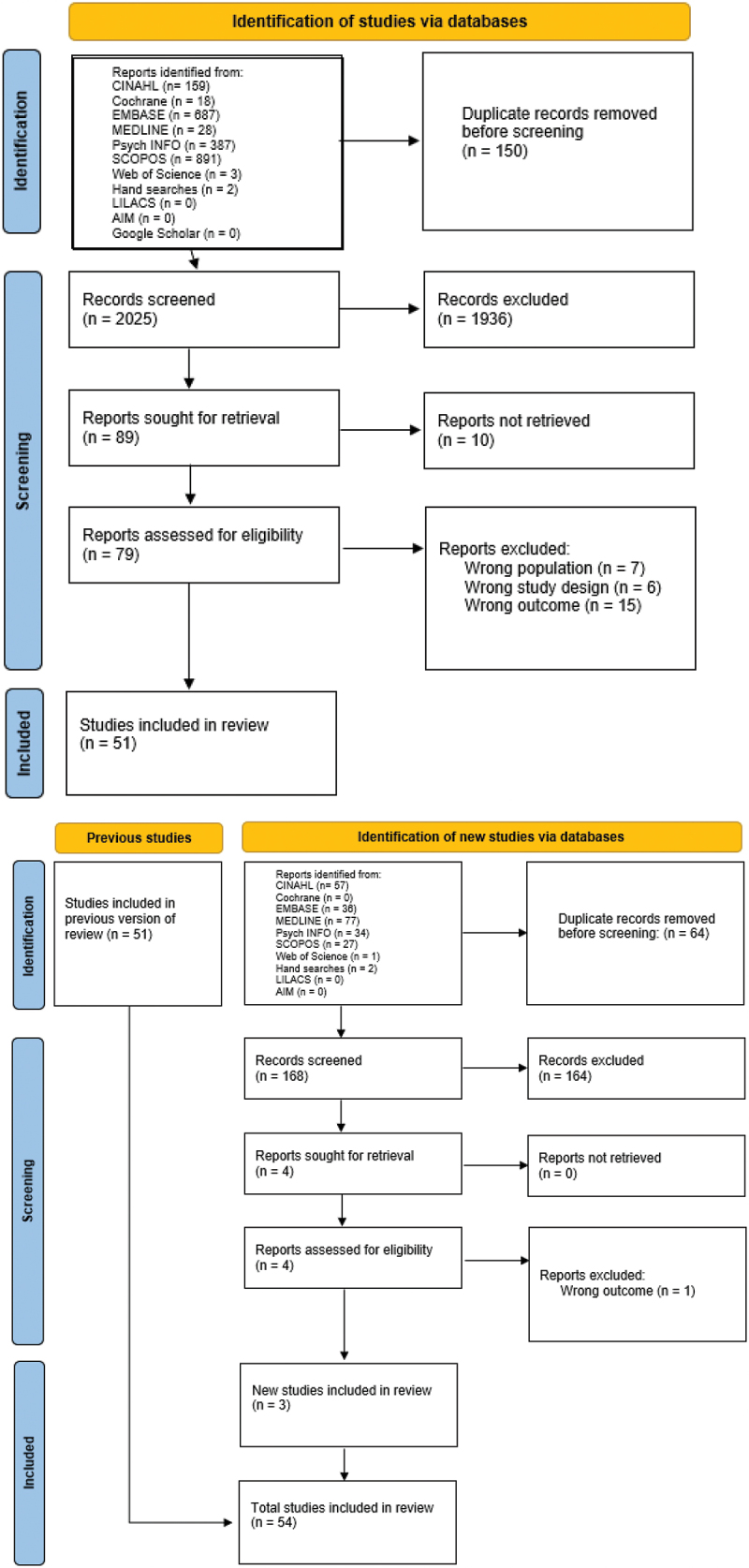
PRISMA diagram of search and inclusion process [25].

Potentially eligible studies were extracted to Endnote20© software and duplicates removed before being uploaded to RAYYAN© software to facilitate screening. Studies were independently screened in two steps (title and abstract screening and full text screening) against the inclusion criteria by two reviewers (KC & AA, MB, or FS). Eleven studies were found to not to be available and we were unable to contact the lead researcher using the details provided in the study abstract. Consequently, these studies were excluded. The number of studies excluded at each stage are presented in Figure 1.

### Quality assessment

The Critical Appraisal Skills Program (CASP) checklist was used to assess the methodological and reporting quality (trustworthiness) of studies included in the QES [26]. Quality was independently assessed by two reviewers (KC & MB). An inter-rater reliability test, Cohen’s Kappa [27], was applied demonstrating substantial agreement in decision making between reviewers. Risk of bias due to limitations in methodological rigour in individual studies was accounted for through application of the CASP checklist, although studies assessed to be low-quality studies were not excluded from the synthesis. The decision not to use quality as an inclusion criterion was made in consideration of the broad range of context in which the studies were conducted [28]. Rather, the CASP checklist was used to facilitate identification of weaknesses in study methodologies and/or reporting and to aid interpretation and assessment of their findings. A sensitivity analysis was later conducted to evaluate the relative impact of quality on the final synthesis findings [29]. A summary narrative table of the quality of individual studies is presented in Appendix B.

### Data extraction

Characteristics of included studies were collated in a bespoke Google Sheet. Variables included author(s), year, country, study design, health setting, cadres of HWs, task shifting focus and key findings of HW perspectives by author(s). The characteristics of the included studies are summarised in Table D1 in Appendix C.

Subsequent data extraction and collation of qualitative findings was undertaken by KC under the supervision of HJ, an experienced qualitative researcher, using NVivo20© software to facilitate data organisation and management. Data extraction was informed by guidelines outlined by Noblit and Hare [30] in which, where possible, first-order constructs defined as direct HW participant quotes were extracted. Therefore, where possible primary data pertaining to HWs perspectives of task shifting were included. However, in studies in which there were insufficient primary data, second-order constructs, defined as authors’ interpretations of participants’ quotes expressed as summaries, were extracted from results and discussion sections of studies to capture all constructs about health worker perspectives of task shifting.

### Data synthesis

Data synthesis followed the method for thematic synthesis outlined by Thomas and Harden [31] who recommend that when a range of epistemological positions are represented across studies synthesised in a review, an epistemology-neutral methodology such as thematic synthesis can be adopted based on a ‘best-fit’ approach to answer the research question. Codes about task shifting were developed and grouped inductively into descriptive themes using a constant-comparative approach. Themes were then synthesised to generate higher-order interpretive themes underpinning facilitators and challenges to task shifting from the perspective of HWs in LMICs. An audit trail recording supporting text for individual themes with originating primary study and decisions made in relation to the inductive development and meaning of themes was kept as synthesis progressed. Maintaining an audit trail contributed to transparency and reproducibility of the review.

## Results

Fifty-four studies (published between 2011 and 2021) with 825 identifiable health worker participants were included in the synthesis (23 studies did not provide a breakdown of numbers of participants drawn from each stakeholder group in the report). The total number of participants from all stakeholder groups across all 54 included studies were between 2394 and 2482. Forty two studies were from sub-Saharan Africa [19,32–71], four from South Asia [72–75], three from South East Asia [18,76,77], one from Central Asia [78] and four were multi-LMIC studies [79–82].

### Study characteristics

Studies located either wholly or partially in sub-Saharan Africa (*n* = 46) predominated, with representation from urban, peri-urban, and rural districts across all studies. Most studies included data from primary healthcare settings (*n* = 51). The perspectives of a wide range of HWs were captured, from senior nurses and doctors, and other SHWs in secondary and tertiary healthcare settings to NSHWs in primary and secondary healthcare settings. The most frequently included cadres of health workers were community-based NSHWs in primary care settings, with most data being obtained through individual interviews and focus group discussions (FGDs). The literature described task shifting happening in many health condition/healthcare settings, with studies specifically referencing task shifting relating to mental health (*n* = 14), HIV/AIDS (*n* = 12) and maternal and child health care (*n* = 11) being most common. The studies described a range of factors stimulating task shifting. Many studies focused on addressing gaps in healthcare provision at national and local levels as a driver for task shifting. Motivating factors in these studies were both to improve patient access to safe and effective healthcare in existing services, and an acknowledgement of unmet needs in the face of increasing demand for healthcare and shortages of SHWs. Whilst many of the studies considered task shifting in the context of specific feasibility or effectiveness trials or health condition initiatives, some realist studies identified task shifting happening informally on an ‘as needed’ basis.

The synthesis generated three analytical themes about what influenced HWs perceptions of task shifting.

### The cultural environment in which task shifting is employed

We defined cultural environments as ‘the workplace’, ‘the local community’ and ‘wider society’. Cultural practices and behavioural norms in any group are shaped by shared values and beliefs and have the potential to exert a strong influence on the acceptability or otherwise on the introduction of new practices, such as when a task shifted healthcare intervention is being proposed. Commonly, where task shifting was viewed to be compatible with achieving the aims of the service, HWs in the workplace found that task shifting enabled them to benefit from supportive, flexible, and positive relationships with colleagues, supervisors, and facility managers. Working together as a team facilitated the practice of task shifting and thus improved access to healthcare for patients.

‘When we requested space for the TASSH (task shifting strategy for hypertension control) sessions, though there was no space, [the director] assured us of his support’. ‘[The director] asked the in-charges of various departments to allow the TASSH team to use their offices’ (two nurses) [45]

We support each other very much—even if you feel there’s pressure, there’s somebody next to you who will grab you and say ‘let’s do it’ … Teamwork is very important. (nurse) [41]

However, misunderstandings about the purpose and scope of task shifting through poor preparation and ineffective communication could result in workplace disharmony and create difficult working conditions. Some SHWs perceived task shifting as diminishing their roles in the health system through endangering their professional status and skills or viewed it as a cost saving exercise in which they could be replaced by less expensive to employ NSHWs.

May be the renewal of prescriptions can be done at the primary facility level, but not the initiation of the treatment. If we come to that, then doctors will have to make their bags and leave Burkina Faso to go to practice elsewhere. I think that is what we should do because if a nurse can do it, doctors will not have their place anymore. (doctor)

Encountering stigmatising discourse and behaviours in the workplace had a detrimental impact on the self-esteem and confidence of NSHWs engaging in task shifting.

There are [staff] in the clinics who are more educated than us. It’s difficult for them to accept us and what we do. It’s difficult for them to accept the change that we bring and it’s hard for them to blend in with us. (lay health worker)

Furthermore, NSHWs reported that when such discriminatory practice was witnessed by patients, this undermined their acceptability as agents of healthcare delivery. Contrary to opening access to healthcare in local communities, this created both a reluctance on the part of HWs to participate in task shifting and on the part of patients to accept healthcare being provided by these cadres.

Considering the influence of local communities and wider society on HW perspectives about task shifting, healthcare services in LMICs are typically organised into vertical healthcare silos with care processes centred around doctors. Consequently, patients and communities may be resistant to accepting healthcare unless provided by a doctor or under their supervision. In primary care settings where gaining access to a doctor is often difficult, NSHWs recruited directly from local communities perceived that sharing a common language, customs and religion acted as a mediator for overcoming this, as they were more likely to be accepted as credible and capable by the community because of their shared background.

They call us Didi [sister]. If anything happens in their own house or in the neighboring houses then they call us. And they trust us so much that if they have any family matter or if their relative has some problem, then they call us. (lay health worker) [75]

However, in some circumstances close ties with the local community were viewed negatively by HWs when engaging in task shifting. This was most notable when the resources required to support healthcare delivery were insufficient or the supply chain was unreliable as with drug stockouts, and in situations such as receiving HIV counselling or mental health support where, due to fear of being stigmatised, maintaining confidentiality was paramount from the perspective of the patient.

Most of them know me as we reside on the area so they were afraid I may divulge their secrets to the wrong people. (community care worker) [39]

In studies including data from secondary and tertiary care settings, sharing a common background was not raised as being either a barrier or enabler to task shifting. This may be because such facilities are usually regionally located with large catchment areas. There is therefore likely to be more diversity in both HW and patient backgrounds. As such, patients will routinely receive health care delivered by HWs, whether specialists or non-specialists, from different backgrounds to their own.

### Access to resources for task shifting

We defined ‘Resources’ as any process or material used to support task shifting. The resources which most strongly influenced HW perceptions of task shifting were training and supervision, materials such as medications and clinic space and remuneration for work undertaken.

HW perspectives on the value of training in preparing them for task shifting were closely linked to how training was developed, resourced, and delivered. Across studies there was much variability in approach to training for task shifting. Comprehensive training packages tailored specifically to develop competent skills in task shifted roles delivered by knowledgeable trainers enabled HWs not only to feel prepared and confident in their extended roles but to feel valued as healthcare providers or as supervisors. However, particularly in those settings where task shifting was practiced informally, training was typically brief and ‘on the job’ for example, receiving verbal instruction or observing a short demonstration.

Learning by doing with others or seeing, actually doing the job. They do not get formal training, on-the-job actually … but they will learn from doing, from mistakes. It is very difﬁcult. (nurse leader)

Closely linked to training, HWs held both positive and negative perceptions of supervision. Relationships with supervisors were held to be important with supervisors who were knowledgeable, empathetic, and readily accessible being especially valued. Supervision was protective of task shifting in that it was seen as a mechanism for empowering HWs to navigate difficulties such as managing the expectations of others with regards to their extended role, developing emotional resilience and confidence and as a means of refreshing and developing condition and intervention skills and knowledge. HWs who encountered difficulties in accessing supervision or whose experience of receiving supervision did not meet the above values were more likely to hold negative perceptions of the impact of task shifting on their working and personal lives. Models of supervision were also an important consideration with value being ascribed to group, peer, and individual sessions dependent upon the focus of the session. Group and peer supervision sessions were valued as an opportunity to learn together and for building supportive peer networks. However, for some, individual sessions were preferred.

I [had] things that I can say but I keep it to myself … I know it is my own fault … Because … everyone was actually so extroverted. (nurse)] [66]

Two considerations that HWs consistently raised as negatively influencing perceptions of task shifting were access to material resources necessary for delivering task shifted work and renumeration and/or incentives for work undertaken. Engaging HWs in task shifting requires access to appropriate material resources. For example, dependent upon the healthcare setting and geographical location these may include a means of transport, suitable clinic space, specialist equipment, specific medications, or health screening kits. Buy-in and leadership from policy makers and facility managers were seen as being crucial in ensuring that provision of necessary resources was both costed and where applicable, supply chains were secured to support task shifting initiatives. Disruptions in access hampered HWs ability to implement task shifting effectively leading to scepticism about its utility.

Lack of commensurate remuneration or other incentives for work undertaken had a strongly negative influence on HWs perceptions about task shifting. This was most marked when task shifting was also associated with taking on heavier workloads and longer working hours. Particularly among lower-paid NSHWs, lack of financial reward or other benefits was associated with a sense of injustice and resentment towards more highly paid colleagues and the healthcare system.

… we do not want to sound like [we] always wants recognition in the form of finance, but that would also help. Because I mean, your load is increasing, your targets are increasing, but money wise it is not increasing. (community-based HIV counsellor) [47]

### Personal values, beliefs, self-efficacy, and emotional resilience

Personal values are the things that people deem to be important to them and provide a framework for how they act. The values that people embrace over time are shaped by experience and interactions with other people, for example, family, religious and community values, education, training, and employment. Beliefs are those things that people believe to be true, the assumptions that they make about the world. Beliefs influence attitudes and actions, such as how a particular action is perceived to benefit the person, other people, or wider society. In the working situation, holding a role that aligns with a person’s values and beliefs fosters self-efficacy and emotional resilience and influences their perceptions of their working role.

HWs’ views about task shifting correlated with their perception of how well it aligned with their values and beliefs with regards to their occupational aspirations. Task shifting created opportunities for professional development, career progression and personal growth through acquisition of new knowledge and skills, mentoring and receiving positive feedback from colleagues, managers, patients, and the wider community. HWs gained confidence and took pride in their work, describing finding their extended roles as rewarding and satisfying.

I love the job. I have that ownership. They say that a doctor is equal to a god. So a lot of people call me “doctor, doctor.” When they say that, we say we are not a doctor. When we say that, but still they say you have helped me so much, that happiness, that feeling we cannot express it. (lay health worker) [75]

Additionally, HWs used their newly acquired knowledge and skills outside of the workplace to enhance personal relationships and for practicing self-care.

It is very important, because before I used to take on the whole world. Now, I know that as I care for my client, I must care for myself, because if I do not care for myself, I am going to burn out. (community-based HIV counsellor) [47]

Task shifting was seen as an opportunity to gain status and respect in the workplace and the community. Increased status could be conferred through gaining a reputation for being able to treat patients successfully and through symbols, such as wearing a uniform and/or badge or being given an enhanced job title. Altruism was strongly motivating, particularly among community-based NSHWs recruited from the same community as their patients. This extended beyond wanting to help individual patients, to increasing access to healthcare and improving the health status of the whole community in which they lived and worked.

If you cure someone who later talks about you and your drugs to another sick person and recommends that he/she pay you a visit because you take good care of people, then you know that they respect you more (community health worker)

Among SHWs, task shifting was positively perceived when viewed as a mechanism for freeing them up from responsibility for undertaking routine healthcare or administrative tasks to focus on more valued and satisfying clinical work.

Where personal values and beliefs were misaligned with the aims of task shifting and/or where difficulties in engaging in task shifting were encountered, HWs expressed negative perceptions of task shifting. Such views and experiences had a detrimental impact on emotional resilience through erosion of self-confidence and sense of control. The struggle to cope was a frequently expressed concern, particularly in anticipation of unmanageable workloads and difficulty in accessing necessary resources to enable them to fulfil their extended role.

We are just overworking ourselves here. Yesterday I left here after 5 pm and reported here very early this morning. I am hungry but there is no where you can go and take lunch or eat. And you can’t even go because the people have lined up out there waiting to be attended to. (nurse) [61]

Less emotionally resilient HWs appeared to employ fewer assertive behaviours to manage expectations of others and conflict within their extended roles. This is particularly pertinent for those HWs working in their own communities who reported struggling to balance internal conflict in being asked to undertake healthcare that fell outside the scope of their remit, but which aligned with their sense of altruism, for example.

According to these health workers, patients sometimes get angry and refuse referrals which in some cases worsen their health conditions and causes death. Therefore, in attempt to meet the expectations of clients, health workers are sometimes forced into handling additional tasks which they are not trained to do (nurses) [61]

## Discussion

Task shifting offers a mechanism for addressing shortfalls in access to healthcare. However, it is a complex process in which careful consideration of multiple factors is required to ensure that HWs are engaged in delivering task shifted healthcare in an effective and sustainable manner. One such factor is how HWs themselves view task shifting as this is likely to influence their willingness to participate in task shifting. Collectively, across cadres of HWs, healthcare setting, and country, the findings of this review showed that where task shifting aligned with personal values, and personally considered support and resource needs, HWs expressed positive views and were more likely to participate in task shifting. Conversely, where these conditions were not met, HWs held negative views and were less committed to task shifting.

HWs spoke about how they evaluated benefits and costs of engaging in task shifting in terms of opportunities for personal and professional growth, personal wellbeing, and occupational security. Being emotionally resilient was an important attribute in being able to navigate the challenges that HWs typically encountered when engaging in task shifting.

How well HWs engaging in task shifting were received in the workplace was a mediator of both positive and negative perceptions of task shifting. Several prior studies have emphasised the role of governance and invested leadership in creating workplace cultures that support task shifting [11,83]. Both centrally and locally derived policies and procedures for task shifting serve to ensure that clarity about its purpose and limitations are clearly communicated within clinical teams to promote collaborative and harmonious working environments, facilitating HWs to develop confidence and self-efficacy in their new roles [40]. Our review determined that in the absence of such policies and procedures as was typically found in settings where an informal culture of task shifting had evolved, some HWs found themselves exposed to inequitable sharing of resources and unethical, exploitative working practices, including being allocated tasks that fell outside their task shifted scope of practice and self-perceived capability on an ‘as-needed’ or ‘convenience’ basis. Spies et al. [64] describe this in terms of a mismatch between task shifting policy and unregulated, opportunistic task shifting. Other authors have also highlighted the association between informal task shifting and unethical practice, most notably affecting cadres of lay health workers [40,84].

Societally, we found that cultural affinity facilitated HW's ability to develop positive patient relationships to deliver effective healthcare. Being recognised for the value of their work in the community was motivating for these HWs who reported taking pride in their work and that it enhanced their status within the community. However, while a previous review [85] has highlighted the benefits of recruiting HWs from local communities, our synthesis found that this was not always a mediator of positive perceptions of task shifting. We found that HWs needed to be emotionally resilient to navigate the boundary between community expectations for meeting health needs and concerns about maintaining confidentiality whilst also working within the bounds of their task shifted roles. Included studies captured how pressure to provide healthcare could be felt externally from the patient, patients’ families, or the community, and that HWs own perceived altruistic duty of care for their community could also be a source of conflict. In keeping with existing literature [17,86], access to resources for training, and supervision, supported by regulatory oversight were identified to be vital for developing emotional resilience necessary for empowering HWs to navigate cultural challenges, thus being mediators of positive perceptions of task shifting. In the absence of these supportive mechanisms task shifting was negatively associated with low status, being at risk of exploitation or causing harm to patients through making mistakes for which HWs could then be held legally accountable.

In our synthesis it was clear that quality and quantity of training was important in determining how HWs felt about task shifting and that training sometimes fell short of their needs. Previous qualitative reviews have highlighted that comprehensive training is a facilitator of task shifting and is associated with job satisfaction and self-efficacy [10,11,87]. However, concerningly, a recent systematic review of task shifted mental health interventions [15] found that training packages placed greatest emphasis on providing intervention-based skills-based training, whilst overlooking that NSHWs had other training needs such as mental health condition knowledge. Other reviews found that training is often insufficient or of inferior quality [11,88]. Whilst acknowledging that other factors such as securing adequate funding and recruiting knowledgeable and skilled trainers influence HWs opinions about the quality and utility of preparatory training received, these findings do also illustrate the importance of engaging with HWs at an early stage in planning training to ensure that their needs are recognised and accommodated.

In keeping with other studies [89,90], we found that access to supportive supervision was at least as important as receiving adequate training in shaping how HWs perceived task shifting. Well considered and accessible supervision was both a mechanism for provision of safe, quality healthcare and for meeting the needs of supervisees. When supervision was difficult to access, inconsistent or more focused on management than support, HWs struggled to cope in their extended roles. Supervision serves two functions: managerial oversight in terms of monitoring fidelity to task shifted practice and productivity, and supportive activities such as professional development, giving training updates, problem-solving, resilience building and providing emotional support. Governance for task shifting also plays an important function in operationalising supervision [10,11]. Supervisory policies both formalise the process of supervision and give weight to its importance. This enables supervisors to have clear objectives in managing supervisees and facilitating professional development. Supervision is also protective for mitigating against burnout and exploitation through empowering HWs to navigate pressures to work beyond their remit and self-determined capabilities, as discussed in relation to cultural influences. Supervision requires the active buy-in of both supervisors and supervisees as well as adequate resources, training, and time. Kemp et al. [91] have previously commented on challenges in translating costed and resourced supervision from task shifting feasibility trials into clinical practice due to shortfalls in funding for healthcare generally and because of workload pressures on both supervisors and supervisees that conflict with making time for regular supervision. SHWs in our synthesis also expressed ambivalence about task shifting into the supervisory roles necessary for the successful roll-out of task shifting initiatives. Earlier studies [92,93] have determined that SHWs perceptions about task shifting are influenced by the degree to which becoming a supervisor aligns with their values and beliefs with regards to their professional identity and proportion of working time spent on patient-facing contacts. To overcome this challenge to task shifting, Spies [92] and Ajuebor et al. [94] propose that pre-registration education programmes and professional registration for nurses and other health professions should include supervision as a core competency for registration and clinical practice to normalise supervisory practice.

Finally, we determined that whilst access to supportive resources for task shifting was an important mediator of perceptions of task shifting, incentives such as renumeration and/or other benefits also played a part in influencing HWs views. Incentives varied across included studies and were notably absent from some studies. Whilst this likely reflects the challenges of costing new health initiatives in already underfunded healthcare systems, it is important to understand the role incentives play in promoting task shifting to HWs. A recent systematic review of studies of non-communicable disease management in LMICs [10] found that none of the 22 studies included a process evaluation to determine the role of incentives and remuneration in facilitating of non-physician health workers to engage in task shifting. A mixed methods review of factors influencing job preferences among HWs providing obstetric care in sub-Saharan Africa [95] found that whilst pay and allowances were positively correlated with employment preferences, for those HWs already commanding salaries above that which enabled them to meet their basic needs, other factors such as access to high-quality human resources management and opportunities for continuing professional development and career progression became more important. This supports that policy makers could adopt a strategic approach to using limited financial resources to attract different cadres of HWs to engage in task shifting.

### Limitations

Identifying studies proved challenging because of variations in terms used to describe task shifting and different cadres of HWs across databases. Although we attempted to develop a comprehensive search strategy, this relied upon authors including a specified term for task shifting and health worker and an LMIC in the study title and/or abstract. We also limited our search strategy to those studies which had an English language abstract. Decisions regarding our search strategy and decision not to include policy documents and reports were made pragmatically, based on available time and human resources. It is therefore possible that some eligible studies and pertinent sources of data were omitted from the QES. However, in keeping with Downe et al. [96], the purpose of the QES was to produce an integrative synthesis of transferable themes consistently found in studies drawn from a wide range of health workers and health care settings in LMICs. We believe, given our sample of 54 included studies representing the perspectives of more than 825 health workers, the review represents a robust, comprehensive synthesis.

## Conclusion

Task shifting healthcare interventions from SHWs to non-specialist HWs has been found to be a clinically and cost-effective solution to addressing shortfalls in human resources to bridge treatment gaps in LMICs. To achieve successful outcomes, endorsement, and engagement by all impacted cadres of HW is an essential element of task shifting. Therefore, to be able to design and deliver effective task shifted healthcare interventions HWs perceptions and experience of task shifting should be considered. This QES brings into focus the complex interplay between the healthcare system, society, and alignment with personal values and beliefs and ability to draw upon personal emotional resilience on influencing HW perspectives of task shifting. Furthermore, it spotlights task shifting governance in protecting and supporting HWs in their extended roles. The findings of this synthesis provide insight into HWs motivation or otherwise to engage in task shifting, which will help to inform interventions for addressing treatment gaps due to shortages in human resources in LMICs. To the best of our knowledge this is the first synthesis of data drawn from studies presenting perspectives across SHWs and NSHWs and drawn from diverse healthcare and country settings in LMICs. The findings of this QES have added to the evidence base of existing knowledge available to assist researchers, policy makers and educators in designing and implementing task shifting health interventions and preparing HWs for task shifting to address treatment gaps in LMICs.

## Supplementary Material

Appendix 4: Table 2-Characteristics of Included Studies

Appendix 3: ENTREQ Statement

Appendix 2: Quality Appraisal

Appendix 1: Search Strategy

## Paper context

Healthcare in LMICs is typically underfunded and under-resourced. Although task shifting has been widely adopted as a method for addressing these challenges, there is limited knowledge from different settings about how task shifting impacts on health workers. Previous studies have found that task shifting poses both occupational opportunities and risks for HWs in LMICs. Bringing together findings from these studies enabled us to identify commonalities in factors across settings that influence HWs perceptions of what the benefits and costs to themselves are when engaging in task shifting. This knowledge is important in helping policy makers to determine how best to engage and support health workers to deliver future task shifted healthcare initiatives to improve access to healthcare in low human resource settings in all LMICs, particularly in settings where currently little knowledge exists.
