# Supplementary material for: Perspectives of health workers engaging in task shifting to deliver health care in low-and-middle-income countries: a qualitative evidence synthesis
Source: Glob Health Action. 2023 Jul 11;16(1):2228112. doi: 10.1080/16549716.2023.2228112 (PMC10337489; doi:10.1080/16549716.2023.2228112)
Supplement: Appendix 4: Table 2-Characteristics of Included Studies [file ZGHA_A_2228112_SM8125.pdf]

APPENDIX 4: Table 2 – Characteristics of Included Studies

Author (year)	Country	Study design	Health setting	Cadres of health workers	Task shifting focus	Form of task shifting	Key findings of health worker perspectives by authors
Abas *et al*, (2016)	Zimbabwe	Case study	Primary	Lay health workers	Mental health	Under supervision	Health workers found the role to be rewarding.
Abrahams-Gessel, *et al* (2015)	Bangladesh, Guatemala, Mexico & South Africa	Mixed methods	Primary	Lay health workers	Cardiovascular disease	Under supervision/ Full delegation after receiving training	Training should be culturally adapted. Challenges such as role definition, career paths and renumeration need to be addressed.
Adler *et al*, (2016)	Ghana	Qualitative	Primary	Healthcare professionals & lay health workers	Hypertension management	Full delegation after receiving training	Cardiovascular nurses and lay health workers reported increased condition awareness and knowledge of how to keep themselves healthy. However, gaps in knowledge led to misrepresentation of clinical guidelines to patients.
Aifah *et al*, (2020)	Nigeria	Concept mapping study	Primary	HIV nurses	Hypertension management	Not applicable	Task shifting requires access to additional resources for condition-specific health education, intervention-specific skills training, counselling skills training.
Angdembe *et al*, (2017)	Nepal	Situational analysis	Primary	Primary healthcare workers & lay health workers	Severe mental disorders	Not applicable	Task shifting requires access to condition specific education and for prescribing medications and training in basic computing and mobile technology together with access to equipment.
Atif *et al*, (2016)	Pakistan	Qualitative	Primary	Primary healthcare workers & peer volunteers	Maternal mental health	Under supervision	Being of the same community and personal characteristics such as empathy and trustworthiness facilitate task shifting. Effective training, supervision, perception of personal gain and endorsement from their families and community enhance peer volunteer motivation. Cultural beliefs and stigma about depression and resistance from mothers/families to receiving treatment from peer volunteers are barriers to task shifting.
Baird *et al*, (2012)	Zambia	Mixed methods	Primary	HIV nurses, clinical officers,	HIV/AIDS	Under supervision	Peer educators can help to address human resource shortfalls in HIV care. Training equips peer educators to provide effective educational

				and community care workers			counselling and medication adherence support. Lack of formal recognition within the healthcare system impacts on peer educator retention.
Bitew *et al,* (2020)	Ethiopia	Qualitative	Primary	Nurses, midwives, health officers and community-based health extension workers	Maternal mental health	Not applicable	Facilitators of task shifting included promoting task shifting in the clinic team, incorporating mental health care into routine primary care delivery, providing mental health training for clinic staff and health extension workers, promoting confidentiality in mental health treatment, and tackling mental health stigma in the community. Barriers to task shifting included patients' perception of breach of confidentiality as the community would know why an extension worker was visiting women patients at home.
Callaghan-Koru *et al*, (2012)	Malawi	Qualitative	Primary	Primary and secondary care health workers	Child health	Under supervision	Task shifting increases access to child healthcare in local communities and reduces workloads at healthcare facilities. Primary care health workers identified the need for adequate resources (training, supervision, equipment). Managerial support, skills acquisition and job satisfaction functioned as motivators to engage in task shifting. Concerns about unmanageable workloads, not meeting community expectations and risks to personal safety functioned as barriers.
Chandhiok *et al*, (2015)	India	Qualitative	Primary and secondary	Skilled birth attendants, state and district level supervisors, medical officers and Aayush practitioners	Maternal	Under supervision	Task shifting could be an effective step in addressing human resource shortfalls in facilitating healthcare facility deliveries in India. Barriers to task shifting – inadequate training, supervision and support and haphazard/lack of acceptance by the healthcare system. Facilitators of task shifting – accreditation system supported by continuing training, supervision, and support.
Cobbing *et al*, (2017)	South Africa	Qualitative	Primary	Community care workers	HIV/AIDS	Peer supervision after receiving training	Community care workers felt that access to contextualized training equipped them with the task shifted skills necessary to deliver HIV care.

APPENDIX 4: Table 2 – Characteristics of Included Studies

Dambisya & Mantinhure, (2012	Uganda	Case study	All levels	Frontline health workers and healthcare students	Multi-sector	Not reported	Task shifting in Uganda is widespread and organized both formally and informally, the main drivers being to address human resource shortfall in the face of high demand for services. Action is being taken to develop top-down policies and procedures for task shifting to offer legal protections and safeguards for health workers where this is being implemented informally and the environment is supportive of task shifting.
Davies *et al*, (2013)	South Africa	Qualitative	Primary	HIV nurses	HIV/AIDS	Under supervision	Quality, safe task shifting relies on well planned and initiated training programs, teamwork, effective communication, ongoing mentoring, and quality assurance however, rapid implementation of the intervention impacted on this.
Dev, Lincoln & Shidaye, (2021)	India	Qualitative	Primary	Community health workers, supervisors, auxiliary nurse midwives and primary care doctors and nurses	Maternal mental health	Peer supervision after receiving training	Factors impacting on community health workers readiness to engage in task shifting included the value ascribed to task shifting, perceived ability to engage in task shifting and value ascribed to their pre-existing job role. Facilitators of task shifting included personal empowerment, gaining respect and trust by the community, professional duty, and relationships with supervisors. Barriers included lack of access to resources.
Druetz *et al*, (2015)	Burkina Faso	Qualitative	Primary	Community health workers	Malaria	Under supervision	Barriers to engaging in task shifting include not feeling valued by the healthcare system and not feeling accountable to their local community.
Ferrinho *et al*, (2012)	Mozambique & Zambia	Qualitative	Primary & secondary	Front line health workers	Multi-sector	Not reported	Task shifting is being widely used in an informal way to address human resource shortfalls in healthcare. Health workers perform tasks outside of their scope of practice taking up much of their available time. Perceived negative impact of task shifting on health workers includes excessive workloads and burnout, lack of access to training, loss of quality in healthcare, risk to patient safety and conflict with patients.

APPENDIX 4: Table 2 – Characteristics of Included Studies

Gajewski *et al*, (2017)	Zambia	Qualitative	Primary & secondary	Surgeons & medical licentiates	Surgery	Under supervision	Task shifting relieves pressure on specialists who value and appreciate the newly trained cadre. Benefits of task shifting include increased job satisfaction, skills acquisition. Challenges to task shifting include lack of recognition by colleagues, unequal status, and lack of renumeration or career path.
Galukande *et al*, (2013)	Uganda	Feasibility analysis	Primary & secondary	Front line health workers	Surgery	Not applicable	Surgical task shifting is commonly practiced informally and in the absence of regulation. Whilst health workers support surgical task shifting in principle to address human resource shortfalls for surgery, in the absence of a regulatory framework the negative impact on health workers include feeling vulnerable/exploited, lack of recognition or renumeration, inadequate training and supervision.
Georgeu *et al*, (2012)	South Africa	Process evaluation	Primary	Nurse trainers & nurses	HIV/AIDS	Under supervision	Facilitators of task shifting – high quality training. Barriers to task shifting – variation in quality and quantity of supervision, inconsistencies in managerial support and access to resources, increased workload, speed of implementation.
Gyamfi *et al*, (2017)	Ghana	Mixed methods	Primary	Community health nurses & enrolled nurses	Hypertension management	Under supervision	Benefits of task shifting include enhanced clinical knowledge and skills, improved people skills. Facilitators of task shifting included quality training. Barriers to task shifting included increased workloads, access to resources.
Gyamfi *et al*, (2020)	Ghana	Qualitative	Primary	Community health nurses	Hypertension management	Under supervision	Facilitators of task shifting include managerial support, mutual respect and trust of colleagues, dedicated time. Barriers to task shifting include access to resources, ongoing training, turnover of managers.
Iwelunmor *et al*, (2019)	Ghana	Realist synthesis	Primary	Community health officers	Hypertension management	Not applicable	Facilitators of task shifting include enhanced clinical knowledge and skills acquisition, ongoing training. Barriers to task shifting include access to resources, poor supervisory relationships, and lack of clinical oversight.
Jacobs *et al*, (2020)	South Africa	Qualitative	Primary	Facility-based counsellors	Mental health	Under supervision	Role play and skills rehearsal in training and using a cascade model of supervision for support

							facilitates successful task shifting. Benefits of task shifting include enabling better treatment outcomes for patients. Barriers included lack of recognition/status among peers and managers, renumeration, access to resources, workload.
Jennings *et al*, (2011)	Benin	Non-inferiority study	Primary	Nurse midwives & ay nurse aides	Maternal	Under supervision	Facilitators to task shifting include helping specialist colleagues, improving health provider relationships, and working together more effectively, being closer to the community with fewer linguistic barriers. Barriers to task shifting included limited time for supervision
Johariyah, Widyawati & Nurdiati, (2020)	Indonesia	Qualitative	Primary	Midwives & community health volunteers	Maternal	Full delegation after receiving training	Facilitators of task shifting include being from the same community.
Knettel *et al*, (2021)	Tanzania	Qualitative	Primary	HIV nurses & Community health workers	HIV/AIDS	Under supervision	Facilitators of task shifting include doing rewarding work and altruism. Barriers to task shifting include patient expectations, lack of recognition.
Kok *et al*, (2020)	Malawi	Qualitative	Primary	Health surveillance assistants	Family planning	Under supervision	Facilitators of task shifting include being from the community, patient trust. Barriers to task shifting include access to resources, unequal status, inconsistent provision of supervision.
Ledikwe *et al*, (2013)	Botswana	Mixed methods	Primary	Doctors, nurses & lay counsellors	HIV/AIDS	Full delegation after receiving training	Facilitators of task shifting include helping the community and valuing their new role. Barriers to task shifting include workload, access to resources, renumeration, job security, career progression
Magidson *et al*, (2019)	South Africa	Qualitative	Primary	HIV adherence counsellor, HIV-TB nurses, physician, clinical medical officer & community health workers	Substance misuse in HIV/AIDS	Full delegation after receiving training	Facilitators to task shifting include being of the same community/ability to relate to patients' situations. Barriers to task shifting insufficient training and supervision, undefined roles and scope of practice, access to resources.
Makhado *et al*, (2020)	South Africa	Qualitative	Primary	Nurses	TB-HIV/AIDS multimorbidity	Full delegation after	Facilitators of task shifting include access to training and ongoing supervision delivered by specialists, defined treatment guidelines. Barriers

						receiving training	to task shifting include relationships with managers and colleagues.
Matsumoto-Takahashi *et al*, (2018)	Philippines	Qualitative	Primary	Community health workers	Malaria control	Not applicable	Facilitators of task shifting include motivation, job satisfaction and enhanced status in the community. Barriers to task shifting include access to resources and renumeration.
Mendenhall *et al*, (2014)	Ethiopia, India, Nepal, South Africa & Uganda	Qualitative	Primary	Primary health workers, Specialists	Mental Health	Not applicable	Key conditions for successful task shifting include recruiting more staff from the local community, access to resources, training, supervision and renumeration.
Mendenhall *et al*, (2018)	Kenya	Qualitative	Primary	Nurses	Mental Health	Not applicable	Facilitators of task shifting included motivation. Barriers included competing priorities, training needs, access to specialists for support, stigma, renumeration, social problems.
Munodawfa, Lund & Schneider, (2017)	South Africa	Process evaluation	Primary	Lay counsellors	Mental Health	Under supervision	Facilitators of task shifting included training and supervision, confidence, and motivation to help the community.
Musyimi *et al*, (2017)	Kenya	Qualitative	Primary	Community health workers	Mental Health	Not applicable	Facilitators of task shifting included motivation and collaboration between health services. Barriers to task shifting included access to resources, patient expectations/mistrust, lack of awareness of task shifting among other health professionals.
Mwangala *et al*, (2015)	Zambia	Qualitative	Secondary	Lay counsellors, nurses & laboratory personnel	HIV/AIDS	Under supervision	Training, supervision, and quality assurance need strengthening tailored to the different cadres of health worker.
Mwisongo *et al*, (2015)	South Africa	Qualitative	Primary	Lay counsellors	HIV/AIDS	Under supervision	Access to adequate and standardized training, access to resources, workload burden, lack of integrative health policy and clear definition of role.
Ngwira *et al*, (2021)	Malawi	Qualitative	Primary	Community health workers	Multi-sector	Not applicable	Facilitators of task shifting included personal resilience, flexibility, teamwork, community engagement, and agency. Barriers to task shifting include access to resources, existing health system structures and gendered-household relations.
Nzinga *et al*, (2019)	Kenya	Qualitative	Secondary	Nurses, support staff & students	Neonatal care	Not reported	Practice of less technical tasks informally delegated to subordinates, often without

							supervision in response to competing priorities and difficult working conditions.
Ochieng *et al*, (2014)	Kenya	Qualitative	Primary	Lay health workers	Multi-sector	Not applicable	Common motivation strategies included training and supportive supervision, access to resources, remuneration, recognition of role and evidence-based community dialogue. In nomadic and peri-urban communities, lay health workers delivered curative services beyond their remit in response to lack of access to health services.
Okyere *et al*, (2017)	Ghana	Qualitative	Primary	Health workers	Multi-sector	Sometimes under supervision	Facilitators of task shifting include working together as a team, flexible approach to taking on "additional tasks" in response to getting work done and patient needs, enhanced self-work and clinical knowledge/skills uplift. Barriers include taking on stressful or "tiresome" tasks, overwork and feeling unprepared.
Pente *et al*, (2021)	Sierra Leone	Qualitative	Primary	Community health workers	Ophthalmic services	Full delegation after receiving training	Facilitators of task shifting included supporting the community and opportunities for career advancement. Barriers included lack of policy implementation and poor health system support for task shifting, impacting on role definition, quality assurance, access to resources and renumeration.
Petersen *et al*, (2011)	South Africa & Uganda	Process evaluation	Primary	Community health workers, nurses, specialist mental health nurses & mental health supervisors	Mental Health	collaborative	Facilitators of task shifting include training, supportive supervision, enhanced condition knowledge and clinical skills. Barriers include workload, competing priorities, mental health stigma, and access to resources.
Smith, (2014)	Malawi	Situational analysis	Primary	Community health workers & supervisors	Multi-sector	Sometimes under supervision	Highlights practice of informal task shifting to cover for human resource shortfalls in health clinics. Health workers and supervisors voiced concerns about access to training, supervision, renumeration, workload, and competing priorities. Health workers perceive their work to be important and of benefit to the population.
Spies, (2016)	Ethiopia, Kenya, Tanzania & Uganda	Qualitative	All levels	Nurse Leaders	Multi-sector	Not applicable	Facilitators of task shifting included tailoring pre-nursing qualification education to prepare nurses for task shifting post-qualification, and comprehensive policy and regulation support for

APPENDIX 4: Table 2 – Characteristics of Included Studies

							task shifting to support and protect nurses engaging in task shifting.
Spies, *et al* (2016)	Uganda	Qualitative	All levels	Nurses	HIV/AIDS	Not applicable	Facilitators of task shifting include nurses' pride in their role. Barriers to task shifting include lack of pre-qualification preparation for task shifting inconsistent/inadequate supervisory support and inadequate policy and regulation.
Tarimo *et al*, (2018)	Tanzania	Mixed methods	Primary	Enrolled nurse/midwives	Maternal & child healthcare	Under supervision	Facilitators of task shifting include team working and commitment to the community. Barriers to task shifting included inadequate training and supervision, lack of resources, meeting community expectations/addressing unmet needs, vulnerability, competing priorities, feeling overworked.
Than *et al*, (2017)	Myanmar	Qualitative	Primary	Community-based auxiliary midwives & midwives	Maternal	Not applicable	Facilitators of task shifting include the capacity to provide improved care during delivery. Barriers to task shifting include inadequate training, and lack of standardized policies and procedures.
van de Water *et al*, (2017)	South Africa	Qualitative	Primary	Nurses	Mental Health	Under supervision	Facilitators of task shifting include supportive supervision and networking. Barriers included access to resources, lone working, and lack of integrated policy for mental health interventions between the health and education sectors.
Vedanthan *et al*, (2016)	Kenya	Qualitative	Primary	Nurses	Hypertension	Not applicable	Facilitators of task shifting included confidence in clinical abilities and hope more a more integrated health system with opportunities for nurses and better outcomes for patients. Barriers included access to resources, inadequate training, work overload and conflicting priorities.
Wall *et al*, (2020)	Kenya	Qualitative	Primary	Lay health workers	Mental Health	Under supervision	Facilitators of task shifting included intrinsic motivation, enhanced condition knowledge and clinical skills positively impacting on professional and personal lives, and supportive supervision. Barriers included work overload and conflicting priorities, stress, and burnout.
Wood *et al*, (2021)	India	Qualitative	Primary	Lay health workers	Mental Health	Under supervision	Barriers and facilitators to task shifting were conceptualized by health workers in relation to three themes, individual, organization, and society.

APPENDIX 4: Table 2 – Characteristics of Included Studies

Yaya Bocoum *et al*, (2013)	Burkina Faso	Case study	All levels	Health workers	HIV/AIDS	Sometimes under supervision	Facilitators of task shifting include team working, congruence between task and personal values, job satisfaction, improved colleague relationships. Barriers included access to training and supportive supervision, professional protectionism, access to resources.
Zimba *et al*, (2021)	Malawi	Qualitative	Secondary	Senior clinicians	Non-communicable disease & depression multimorbidity	Not applicable	Facilitators to task shifting include helping patients/improving access to mental health treatment, team working. Barriers included lack of condition knowledge and clinical skills, access to training, access to resources, increased workload, lack of policy and procedures for task shifting.

10.1080/16549716.2023.2228112
