# Supplementary material for: Perspectives of health workers engaging in task shifting to deliver health care in low-and-middle-income countries: a qualitative evidence synthesis
Source: Glob Health Action. 2023 Jul 11;16(1):2228112. doi: 10.1080/16549716.2023.2228112 (PMC10337489; doi:10.1080/16549716.2023.2228112)
Supplement: Appendix 1: Search Strategy [file ZGHA_A_2228112_SM5952.docx]

| **#** | **Query** | **Limiters/Expanders** | **Last Run Via** | **Results** |
| --- | --- | --- | --- | --- |
| S50 | S46 AND S47 | Expanders - Apply equivalent subjects Search modes Boolean/Phrase | Interface - EBSCOhost  Research Databases  Search Screen - Advanced  Search  Database - CINAHL Complete | Display |
| S49 | S45 AND S47 | Expanders - Apply equivalent subjects Search modes Boolean/Phrase | Interface - EBSCOhost  Research Databases  Search Screen - Advanced  Search  Database - CINAHL Complete | Display |
| S48 | S30 AND S47 | Expanders - Apply equivalent subjects Search modes Boolean/Phrase | Interface - EBSCOhost  Research Databases  Search Screen - Advanced  Search  Database - CINAHL Complete | Display |
| S47 | (“low- and middle-income countries” OR LMIC’s OR “S* Asia*” OR Africa* OR  “Sub-Saharan Africa*”  OR “S* America*” OR  “Latin America*” OR “C* America” OR Caribbean  OR Afghanistan OR  Albania OR Algeria OR  “American Samoa” OR  Angola OR Argentina OR  “Argentine Republic” OR  Armenia OR Azerbaijan  OR Bangladesh OR  Belarus OR Byelarus OR  Belorussia OR Belize OR  Benin OR Bhutan OR  Bolivia OR Bosnia OR  Botswana OR Brazil OR  Bulgaria OR Burma OR  “Burkina Faso” OR  Burundi OR “Cabo Verde”  OR “Cape Verde” OR  Cambodia OR Cameroon OR Central AfricanRepublic OR Chad OR China OR Colombia OR Comoros OR Comores OR Comoro OR Congo OR Costa Rica OR “Cote dI’Voire OR Cuba OR Djibouti OR Dominica OR “Dominican Republic” OR Ecuador OR Egypt OR “El Salvador” OR Fifi OR Gabon OR Gambia OR Gaza OR “Georgia Republic” OR Georgian OR Ghana OR Grenada OR Grenadines OR Guatemala OR Guinea OR “Guinea Bissau” OR Guyana OR Haiti OR Herzegovinia OR Hercegovina OR Honduras OR India OR Indonisia OR Iran OR Iraq OR Jamaica OR Jordan OR Kazakhstan OR Kenya OR Kiribati OR Korea OR Kosovo OR Kyrgyz OR Kirghizia OR Kirghiz OR Kirgistan OR Kyrgyzstan OR “Lao PDR” OR Laos OR Lebanon OR Lesotho OR Liberia OR Libya OR Macedonia OR Madagacar OR Malawi OR Malay OR Malaya OR Malaysia OR Maldives OR Mali OR “Marshall Islands” OR Mauritania OR Mauritius OR Mexico OR Micronesia OR Moldova OR Mongolia OR Montenegro OR Morocco OR Mozambique OR Myanmar OR Namibia OR Nauru OR Nepal OR Nicaragua OR Niger OR Nigeria OR Pakistan OR Palau OR Panama OR “Papua New Guinea” or Paraguay OR Peru OR Philippines OR Phillippines OR Philipines OR Phillipines OR Principe OR Romania OR Rwanda OR Ruanda OR Samoa OR “Sao Tome” OR Senegal OR Serbia OR “Sierra Leone” OR “Solomon Islands” OR Somalia OR “South Africa” OR “South Sudan” OR “Sri Lanka” OR “St Lucia” OR “St Vincent”OR Sudan” OR Surinam OR Suriname OR Swaziland OR Syria OR “Syrian Arab Republic” OR Tajikstan OR Tadzhikstan OR Tadzhik OR Tanzania OR Thailand OR Timor OR Togo OR Tonga OR Tunisia OR Turkey OR Turkmen OR Turkmenistan OR Tuvalu OR Uganda OR Ukraine OR Uzbek OR Uzbekistan OR Vanuatu OR Venezuela OR Vietnam OR “West Bank” OR Yemen OR Zambia OR Zimbabwe) | Expanders - Apply equivalent subjects Search modes Boolean/Phrase | Interface - EBSCOhost  Research Databases  Search Screen - Advanced  Search  Database - CINAHL Complete | Display |
| S46 | S30 AND S45 | Expanders - Apply equivalent subjects Search modes Boolean/Phrase | Interface - EBSCOhost  Research Databases  Search Screen - Advanced  Search  Database - CINAHL Complete | Display |
| S45 | S31 OR S32 OR S33 OR  S34 OR S35 OR S36 OR S37 OR S38 OR S39 OR S40 OR S41 OR S42 OR  S43 OR S44 | Expanders - Apply equivalent subjects | Interface - EBSCOhost  Research Databases  Search Screen - Advanced  Search  Database - CINAHL Complete | Display |
| S44 | work adj3 substitut* | Expanders - Apply equivalent subjects Search modes Boolean/Phrase | Interface - EBSCOhost  Research Databases  Search Screen - Advanced  Search  Database - CINAHL Complete | Display |
| S43 | “work substitut*” | Expanders - Apply equivalent subjects Search modes Boolean/Phrase | Interface - EBSCOhost  Research Databases  Search Screen - Advanced  Search  Database - CINAHL Complete | Display |
| S42 | task* adj3 substitut* | Expanders - Apply equivalent subjects Search modes Boolean/Phrase | Interface - EBSCOhost  Research Databases  Search Screen - Advanced  Search  Database - CINAHL Complete | Display |
| S41 | “task substitut*” | Expanders - Apply equivalent subjects Search modes Boolean/Phrase | Interface - EBSCOhost  Research Databases  Search Screen - Advanced  Search  Database - CINAHL Complete | Display |
| S40 | work adj3 optimis | Expanders - Apply equivalent subjects Search modes Boolean/Phrase | Interface - EBSCOhost  Research Databases  Search Screen - Advanced  Search  Database - CINAHL Complete | Display |
| S39 | “work optimis*” | Expanders - Apply equivalent subjects Search modes Boolean/Phrase | Interface - EBSCOhost  Research Databases  Search Screen - Advanced  Search  Database - CINAHL Complete | Display |
| S38 | task* adj3 optimis | Expanders - Apply equivalent subjects Search modes Boolean/Phrase | Interface - EBSCOhost  Research Databases  Search Screen - Advanced  Search  Database - CINAHL Complete | Display |
| S37 | “task optimis*” | Expanders - Apply equivalent subjects Search modes Boolean/Phrase | Interface - EBSCOhost  Research Databases  Search Screen - Advanced  Search  Database - CINAHL Complete | Display |
| S36 | work adj3 delegat* | Expanders - Apply equivalent subjects Search modes Boolean/Phrase | Interface - EBSCOhost  Research Databases  Search Screen - Advanced  Search  Database - CINAHL Complete | Display |
| S35 | “work delegat*” | Expanders - Apply equivalent subjects Search modes Boolean/Phrase | Interface - EBSCOhost  Research Databases  Search Screen - Advanced  Search  Database - CINAHL Complete | Display |
| S34 | task* adj3 shar*" | Expanders - Apply equivalent subjects Search modes Boolean/Phrase | Interface - EBSCOhost  Research Databases  Search Screen - Advanced  Search  Database - CINAHL Complete | Display |
| S33 | "task shar*" | Expanders - Apply equivalent subjects Search modes Boolean/Phrase | Interface - EBSCOhost  Research Databases  Search Screen - Advanced  Search  Database - CINAHL Complete | Display |
| S32 | "task* adj3 shift*" | Expanders - Apply equivalent subjects Search modes Boolean/Phrase | Interface - EBSCOhost  Research Databases  Search Screen - Advanced  Search  Database - CINAHL Complete | Display |
| S31 | "task shift*" | Expanders - Apply equivalent subjects Search modes Boolean/Phrase | Interface - EBSCOhost  Research Databases  Search Screen - Advanced  Search  Database - CINAHL Complete | Display |
| S30 | S1 OR S2 OR S3 OR S4  OR S5 OR S6 OR S7 OR  S8 OR S9 OR S10 OR  S11 OR S12 OR S13 OR  S14 OR S15 OR S16 OR  S17 OR S18 OR S19 OR  S20 OR S21 OR S22 OR  S23 OR S24 OR S25 OR S26 OR S27 OR S28 OR  S29 | Expanders - Apply equivalent subjects Search modes Boolean/Phrase | Interface - EBSCOhost  Research Databases  Search Screen - Advanced  Search  Database - CINAHL Complete | Display |
| S29 | "traditional healer" | Expanders - Apply equivalent subjects Search modes Boolean/Phrase | Interface - EBSCOhost  Research Databases  Search Screen - Advanced  Search  Database - CINAHL Complete | Display |
| S28 | "barefoot doctor" | Expanders - Apply equivalent subjects Search modes Boolean/Phrase | Interface - EBSCOhost  Research Databases  Search Screen - Advanced  Search  Database - CINAHL Complete | Display |
| S27 | "community health aid*" | Expanders - Apply equivalent subjects Search modes Boolean/Phrase | Interface - EBSCOhost  Research Databases  Search Screen - Advanced  Search  Database - CINAHL Complete | Display |
| S26 | “community based  distributo*” | Expanders - Apply equivalent subjects Search modes Boolean/Phrase | Interface - EBSCOhost  Research Databases  Search Screen - Advanced  Search  Database - CINAHL Complete | Display |
| S25 | "village health worker*" | Expanders - Apply equivalent subjects Search modes Boolean/Phrase | Interface - EBSCOhost  Research Databases  Search Screen - Advanced  Search  Database - CINAHL Complete | Display |
| S24 | "community educator*" | Expanders - Apply equivalent subjects Search modes Boolean/Phrase | Interface - EBSCOhost  Research Databases  Search Screen - Advanced  Search  Database - CINAHL Complete | Display |
| S23 | "community health volunteer*" | Expanders - Apply equivalent subjects Search modes Boolean/Phrase | Interface - EBSCOhost  Research Databases  Search Screen - Advanced  Search  Database - CINAHL Complete | Display |
| S22 | "consejer*" | Expanders - Apply equivalent subjects Search modes Boolean/Phrase | Interface - EBSCOhost  Research Databases  Search Screen - Advanced  Search  Database - CINAHL Complete | Display |
| S21 | “natural helper*” | Expanders - Apply equivalent subjects Search modes Boolean/Phrase | Interface - EBSCOhost  Research Databases  Search Screen - Advanced  Search  Database - CINAHL Complete | Display |
| S20 | “peer educator*” | Expanders - Apply equivalent subjects Search modes Boolean/Phrase | Interface - EBSCOhost  Research Databases  Search Screen - Advanced  Search  Database - CINAHL Complete | Display |
| S19 | “peer worker*” | Expanders - Apply equivalent subjects Search modes Boolean/Phrase | Interface - EBSCOhost  Research Databases  Search Screen - Advanced  Search  Database - CINAHL Complete | Display |
| S18 | “community health advisor*” | Expanders - Apply equivalent subjects Search modes Boolean/Phrase | Interface - EBSCOhost  Research Databases  Search Screen - Advanced  Search  Database - CINAHL Complete | Display |
| S17 | “lay facilitator*” | Expanders - Apply equivalent subjects Search modes Boolean/Phrase | Interface - EBSCOhost  Research Databases  Search Screen - Advanced  Search  Database - CINAHL Complete | Display |
| S16 | “indigenous volunteer*” | Expanders - Apply equivalent subjects Search modes Boolean/Phrase | Interface - EBSCOhost  Research Databases  Search Screen - Advanced  Search  Database - CINAHL Complete | Display |
| S15 | "lay counsel*" | Expanders - Apply equivalent subjects Search modes Boolean/Phrase | Interface - EBSCOhost  Research Databases  Search Screen - Advanced  Search  Database - CINAHL Complete | Display |
| S14 | "lay educato*" | Expanders - Apply equivalent subjects Search modes Boolean/Phrase | Interface - EBSCOhost  Research Databases  Search Screen - Advanced  Search  Database - CINAHL Complete | Display |
| S13 | "lay advisor*" | Expanders - Apply equivalent subjects Search modes Boolean/Phrase | Interface - EBSCOhost  Research Databases  Search Screen - Advanced  Search  Database - CINAHL Complete | Display |
| S12 | "lay worker*" | Expanders - Apply equivalent subjects Search modes Boolean/Phrase | Interface - EBSCOhost  Research Databases  Search Screen - Advanced  Search  Database - CINAHL Complete | Display |
| S11 | "lay health worker*" | Expanders - Apply equivalent subjects Search modes Boolean/Phrase | Interface - EBSCOhost  Research Databases  Search Screen - Advanced  Search  Database - CINAHL Complete | Display |
| S10 | "lay advocate*" | Expanders - Apply equivalent subjects Search modes Boolean/Phrase | Interface - EBSCOhost  Research Databases  Search Screen - Advanced  Search  Database - CINAHL Complete | Display |
| S9 | abulea* | Expanders - Apply equivalent subjects Search modes Boolean/Phrase | Interface - EBSCOhost  Research Databases  Search Screen - Advanced  Search  Database - CINAHL Complete | Display |
| S8 | promotore* | Expanders - Apply equivalent subjects Search modes Boolean/Phrase | Interface - EBSCOhost  Research Databases  Search Screen - Advanced  Search  Database - CINAHL Complete | Display |
| S7 | "nurse* aid*" | Expanders - Apply equivalent subjects Search modes Boolean/Phrase | Interface - EBSCOhost  Research Databases  Search Screen - Advanced  Search  Database - CINAHL Complete | Display |
| S6 | nurse* | Expanders - Apply equivalent subjects Search modes Boolean/Phrase | Interface - EBSCOhost  Research Databases  Search Screen - Advanced  Search  Database - CINAHL Complete | Display |
| S5 | counsel* | Expanders - Apply equivalent subjects Search modes Boolean/Phrase | Interface - EBSCOhost  Research Databases  Search Screen - Advanced  Search  Database - CINAHL Complete | Display |
| S4 | “facility-based-counsel*” | Expanders - Apply equivalent subjects Search modes Boolean/Phrase | Interface - EBSCOhost  Research Databases  Search Screen - Advanced  Search  Database - CINAHL Complete | Display |
| S3 | "community health worker*" | Expanders - Apply equivalent subjects Search modes Boolean/Phrase | Interface - EBSCOhost  Research Databases  Search Screen - Advanced  Search  Database - CINAHL Complete | Display |
| S2 | "non-specialist health worker*" | Expanders - Apply equivalent subjects Search modes Boolean/Phrase | Interface - EBSCOhost  Research Databases  Search Screen - Advanced  Search  Database - CINAHL Complete | Display |
| S1 | "health worker*" | Expanders - Apply equivalent subjects Search modes Boolean/Phrase | Interface - EBSCOhost  Research Databases  Search Screen - Advanced  Search  Database - CINAHL Complete | Display |
